# Bioengineering *Comamonas testosteroni* CNB-1: a robust whole-cell biocatalyst for efficient PET microplastic degradation

**DOI:** 10.1186/s40643-023-00715-7

**Published:** 2023-12-18

**Authors:** Zhanqing Cao, Wei Xia, Shilei Wu, Jiale Ma, Xiaoli Zhou, Xiujuan Qian, Anming Xu, Weiliang Dong, Min Jiang

**Affiliations:** 1 College of Biotechnology and Pharmaceutical Engineering, Nanjing Tech University, Nanjing, 211816 China; 2 State Key Laboratory of Materials-Oriented Chemical Engineering, Nanjing Tech University, Nanjing, 211816 China; 3 Key Laboratory for Waste Plastics Biocatalytic Degradation and Recycling, Nanjing Tech University, Nanjing, 211816 China

## Abstract

The escalating crisis of polyethylene terephthalate (PET) microplastic contamination in biological wastewater treatment systems is a pressing environmental concern. These microplastics inevitably accumulate in sewage sludge due to the absence of effective removal technologies. Addressing this urgent issue, this study introduces a novel approach using DuraPETase, a potent enzyme with enhanced PET hydrolytic activity at ambient temperatures. Remarkably, this enzyme was successfully secreted from *Comamonas testosteroni* CNB-1, a dominant species in the active sludge. The secreted DuraPETase showed significant hydrolytic activity toward *p*-NPB and PET nanoplastics. Furthermore, the CNB-1 derived whole-cell biocatalyst was able to depolymerize PET microplastics under ambient temperature, achieving a degradation efficiency of 9% within 7 days. The CNB-1-based whole biocatalysts were also capable of utilizing PET degradation intermediates, such as terephthalic acid (TPA) and ethylene glycol (EG), and bis(2-hydroxyethyl)-TPA (BHET), for growth. This indicates that it can completely mineralize PET, as opposed to merely breaking it down into smaller molecules. This research highlights the potential of activated sludge as a potent source for insitu microplastic removal.

**Graphical Abstract:**

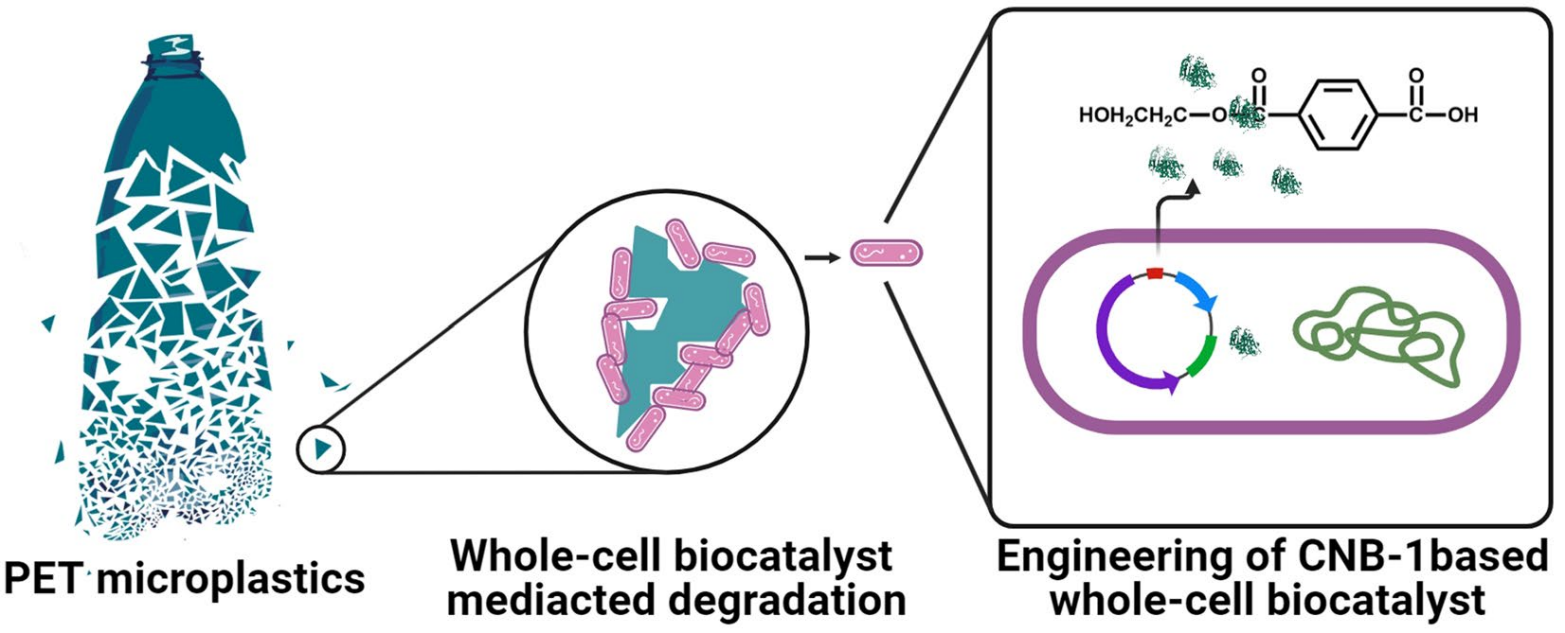

## Introduction

Plastics have revolutionized modern life, but the accumulation of synthetic polymers in landfills and the environment has created a global pollution crisis (Xu et al. 2023). Additionally, there has been increasing research interest in the potential adverse effects of microplastics (ranging from 1 μm to 5 mm) on ecosystems and human health (Wei et al. 2019). Microplastics are produced through the weathering and mechanical tearing (such as tires) of larger pieces of plastic, and are also directly manufactured and utilized in many personal care and cosmetic products (PCCPs) (Nizzetto et al. 2016; van der Laan et al. 2023). Furthermore, microplastics are transported from raw wastewater to wastewater treatment plants (WWTPs) (Browne et al. 2013; Sun et al. 2020), WWTPs can efficient remove over 90% of microplastics from raw wastewater (Carr et al. 2016). However, duo to this high removal efficiency, most microplastics end up being retained in the sewage sludge (Mahon et al. 2017; Zhang et al. 2020b).

Microplastics are often highly resistant to microbial degradation due to their chemically inert backbone structure (Chen et al. 2022; Thakur et al. 2023). Consequently, the fast release of microplastics, coupled with their strong resistance to degradation, leads to the rapid accumulation of these particles in the sewage sludge. Studies have indicated that concentrations of microplastics in the sludge samples ranged from 1.6–56.4 × 10^3^ particles per kilogram of dry sludge (Li et al. 2018). This microplastic-rich sludge is often further processed either as landfilled or used as farmland fertilizer and can re-enter the water system through soil erosion or surface runoff (Nizzetto et al. 2016). Despite the application of various treatments, such as aerobic digestion and industrial composting, to remove harmful substances from sludge before agriculture applications (Mahon et al. 2017), current technologies remain inadequate for the complete elimination of microplastics (Li et al. 2022; Mahon et al. 2017). Therefore, the development of efficient and environmentally friendly methods, such as in situ microbial degradation of microplastics in WWTPs and sludge, is of great importance in preventing further migration of microplastics into the environment.

Polyvinyl chloride (PVC), polystyrene (PS), polypropylene (PP), and polyethylene terephthalate (PET) are the most common microplastics found in sludges (Rochman et al. 2013). Among them, the biodegradation of PET into monomers is has been proven feasible (Xu et al. 2023). An extraordinary finding was the discovery of the bacterium *Ideonella sakaiensis* 201-F6, in which *Is*PETase and MHETase were biochemically characterized to work together to degrade low-crystalline PET into monomers (Knott et al. 2020; Yoshida et al. 2016). As other successful polyester hydrolase examples, leaf and branch compost cutinase (LCC), *Thermobifida fusca* cutinase (TfH), and recently discovered PE-H, PHL-7, MG8 were also found from different microorganisms or metagenomic DNA extracts (Dong et al. 2020; Xu et al. 2023). A prominent example is DuraPETase (Cui et al. 2021), which was obtained after the mutation of ten amino acids in wild-type *Is*PETase (Cui et al. 2021). The catalysis mechanism of DuraPETase involves the hydrolysis of the ester bond within the PET molecule (Cui et al. 2021). Compared to the wild-type *Is*PETase, DuraPETase showed over 300-fold higher hydrolytic activity against PET powder (Cui et al. 2021). These PET hydrolases have inspired the development of functional PET degrading strains for in situ degradation of PET microplastics within the activated sludge system.

*Comamonadaceae* is a dominant species in the activated sludge system, comprising more than 2% of the total abundance of sludge microorganisms (Wu et al. 2019; Zhang et al. 2020a). Additionally, studies have reported that *Comamonas* can degrade terephthalic acid (TPA) and ethylene glycol (EG), which are the main products of PET depolymerization (Aksu et al. 2021; Dierkes Robert et al. 2022; Hosaka et al. 2013; Ma et al. 2009). The objective of this study is to create a whole-cell biocatalyst for the degradation of PET microplastics. To achieve this, DuraPETase was heterologous expressed and optimized in *Comamonas testosteroni* CNB-1. This strain was isolated from a biological reactor treating wastewater from a *p*-chloronitrobenzene production factory (Liu et al. 2007; Wang et al. 2021). The PET-degrading biocatalyst derived from CNB-1 demonstrated significant degradation activity against Impranil^®^ DLN and PET nanoparticles. This degradation ability was further confirmed by scanning electron microscopy (SEM) images of PET film surfaces after 14 days of degradation. Moreover, in relevance to application scenarios, the capability of CNB-1 derived PET-degrading biocatalyst to PET microplastics was further demonstrated. This study has opened up avenues for developing environmentally friendly biocatalytic approaches to address microplastic accumulation, and highlights the potential application of CNB-1 derived PET-degrading biocatalyst in sludge microplastic degradation.

## Material and methods

### Bacterial strains, plasmids, and culture conditions

*C. testosteroni* CNB-1(CGMCC 1.12282) was cultivated and maintained at 30 °C in LB broth or on LB plates with 1.5% (wt/vol) agar. For PET degradation, a mineral salt medium (MSM, pH 7.0) containing (NH_4_)_2_SO_4_ 1 g/L, K_2_HPO_4_ 6 g/L, KH_2_PO_4_ 1 g/L, MgSO_4_·7H_2_O 0.1 g/L, NaCl 5 g/L was used by adding PET nanoplastics or microplastics. The pET-29a(+) vector served as the backbone for expressing enzymes (*Is*PETase, LCC, and DuraPETase) in *E. coli* BL21(DE3). For the overexpression of DuaPETase in CNB-1, the plasmid used was a derivative of pBBR1MCS-2 (pBBR1MCS2pfer), with its promoter replaced with a strong promoter from *C. testosteroni* CNB-1 (Huang et al. 2019). When necessary, antibiotics were added at the following final concentrations: 200 μg/ml kanamycin for *C. testosteroni*; 50 μg/ml kanamycin, and 100 μg/ml ampicillin for *E. coli*.

### Expression of DuraPETase in *C. testosteroni*

DuraPETase (Genbank: GAP38373.1), which was mutated of 10 amino acid sites (S214H/I168R/W159H/S188Q/R280A/A180I/G165A/Q119Y/L117F/T140D) in the backbone of *Is*PETase. In this study, the ten mutated sites were regenerated in *Is*PETase and then commercially synthesized with codon optimization for expression in *E. coli* cells (GenScript, Nanjing, China). The genes were cloned into the pBBR1MCS2pfer vector, a pBBR1MCS-2 derivative plasmid whose promoter was replaced with a strong promoter from *C. testosteroni*, and contained a *gfp* gene (Huang et al. 2019). DuraPETase was inserted before the *gfp* gene to help further evaluate the expression via GFP intensity. To help the secretion of DuraPETase, an endogenic secreted signaling peptide OmpC (from CNB-1, gene locus: CtCNB1_1477) was tagged before DuraPETase, generating the final plasmid, pBBRpfer-OmpC-DuraPETase-GFP.

For plasmids transformation, *C. testosteroni* was prepared for electrocompetent cells by washing twice with ice-cold 10% glycerol, and then concentrated 100-fold. Electroporation was performed with prechilled 2 mm gap electroporation cuvettes (Bio-Rad) and electroporated at 2.5 kV with a Bio-Rad MicroPulser. 1 mL of LB was added to shocked cells and recovered for 2 h, before plating on LB agar with appropriate antibiotics. The green fluorescent signal was measured using the Synergy H4 Hybrid Reader (BioTek). To determine the total GFP intensity, 200 μL of overnight culture was collected in a 96-well plate for recording. The extracellular GFP was subsequently measured from the supernatant after centrifugation.

### Extracellular hydrolase activity measurement by *p*-nitrophenyl butyrate (*p*-NPB) assay

The hydrolase activity of CNB-1/pDuraPETase was evaluated using *p*-NPB (Sigma-Aldrich N9876) as a model substrate with certain modifications. CNB-1/pDuraPETase was incubated in MSM medium containing 5% (v/v) LB for 3 days at 30 °C under aerobic conditions, cells were harvested by centrifugation at 7000*g* for 2 min, and the supernatant was used for measurement of the extracellular hydrolase activity. Absorbance assays were carried out on the Tecan plate reader, using 200 μL reactions composed of 20 μL supernatant and 10 μL 1 M *p*-NPB in Tris buffer (pH 8.0) (Ateşlier and Metin 2006; Qiao et al. 2022). The production of *p*-nitrophenol (*p*-NP) was monitored at 405 nm within 30 min at 30 °C. One unit of activity (1 U) was defined as the enzymatic production of 1 μmol *p*-NP per min at 30 °C.

### HPLC analysis of the degradation product of PET

HPLC was performed on an Agilent 1260 HPLC System using a C18 Reversed-phase column (4.6 mm × 250 mm, Agilent Technologies). The mobile phase was methanol/18 mM phosphate buffer (pH 2.5) at a flow rate of 0.5 mL min^−1^, and the effluent was monitored at a wavelength of 240 nm. The typical elution conditions were as follows: 0 to 30 min, 25% (v/v) methanol, and 30 to 50 min, 25–100% methanol linear gradient. 10 μL of the final degradation products was used for detection.

Standard mono(2-hydroxyethyl)-TPA (MHET) was obtained from the complete hydrolysis using LCC of bis(2-hydroxyethyl)-TPA (BHET). After the complete hydrolysis of BHET to MHET was confirmed by HPLC, the protein removed from the reaction mixture with Amicon Ultra 10 kDa (Merck Millipore). Then, MHET, TPA, and BHET of different concentrations were used to run HPLC, and the standard curves of the three were drawn according to the relationship between peak area and sample loading.

### Thermogravimetric analysis for PET nanoplastics

PET nanoplastics were produced as previously described with slight modifications (Wei et al. 2014). Briefly, 0.1 g of PET films with an initial crystallinity of approximately 4% was dissolved in 10 mL 1,1,1,3,3,3-hexafluoro-2-propanol (HFIP) at room temperature for at least 12 h. This solution was dropped into 100 mL ultra-filtered water while stirring thoroughly at 10,000 rpm using an overhead stirrer. Precipitated PET formed small particles, which can preserve as stable aqueous suspension whereas the larger aggregates were removed with a standard folded filter. The organic solvent was then removed from the particle suspension using a rotary evaporator. For PET nanoplastics degradation, bacteria were grown in LB overnight and then inoculated by 100-times dilution into 50 mL fresh MSM media, and 15 mL of the prepared PET nanoplastics was added for incubation at 30 °C for 10 days.

Thermogravimetric analysis was used to determine the degradation of PET nanoplastics by CNB-1 derived PET-degrading biocatalyst. The experiments were performed on HY4520 Instruments under nitrogen with a heating rate of 20 °C/min from room temperature up to 800 °C. The mass loss with time and temperature is monitored. PET nanoplastics with or without CNB-1 derived PET-degrading biocatalysts were assessed for their PET nanoplastic degradation.

### PET microplastic degradation and weight loss measurement

The amorphous Goodfellow film (crystallinity of 4%) (Chen et al. 2021) was cut into 2 × 2 mm fragments. These small fragments were then smashed and passed through a 40-mesh sieve to obtain PET microplastic particles with an average size of approximately 425 μm, and a crystallinity around 10%. To prepare the microplastics for use in the degradation experiment, the PET microplastics were washed twice with 75% ethanol, followed by sterile water. 100 mg of the prepared microplastics was added into 100 mL for the degradation experiment mixture. In parallel, bacteria were grown overnight in LB media, and then inoculated by 100-times dilution into fresh MSM media containing 5% (v/v) LB, and incubated at 30 °C for 7 days. Three technical replicates were performed for each strain. To facilitate the efficient removal of biological tissues from PET microplastics and achieve accurate weight loss measurements after degradation, the PET microplastics were treated with a 10% KOH solution and subsequently incubated at 60 °C for 24 h. This process successfully digested and removed all biological tissues present on the PET microplastics.

### SEM analysis

At the end of the PET film degradation (14 days), the reactions were stopped by adding 5 mL of 3 M HCl. The PET pieces in the flasks were used for scanning electron microscopy (SEM) analysis by SU-8100 (Hitachi, Japan) after washing with 1% SDS, ultrapure water, and ethanol, respectively. In SEM analysis, samples were coated with Au for 180 s at 20 mA and were scanned under a low vacuum at 10 kV.

## Results and discussion

### DuraPETase is suitable for heterologous expression in *C. testosteroni* CNB-1

For the heterologous expression of PET hydrolyses in *C. testosteroni* CNB-1, we initially compared the activity of several previously reported enzymes including *Is*PETase, DuraPETase, and LCC, at a temperature of 30 °C. This selection was made because the optimum growth temperature for the CNB-1 strain is 30 °C, and the typical wastewater treatment process is also conducted at a similar temperature. While all three enzymes exhibited PET degradation activity, it has been reported that DuraPETase and LCC demonstrate to have good activity at temperatures above 60 °C (Cui et al. 2021; Tournier et al. 2020), whereas *Is*PETase has relatively low catalysis activity at 30 °C (Knott et al. 2020).

**Fig. 1:**
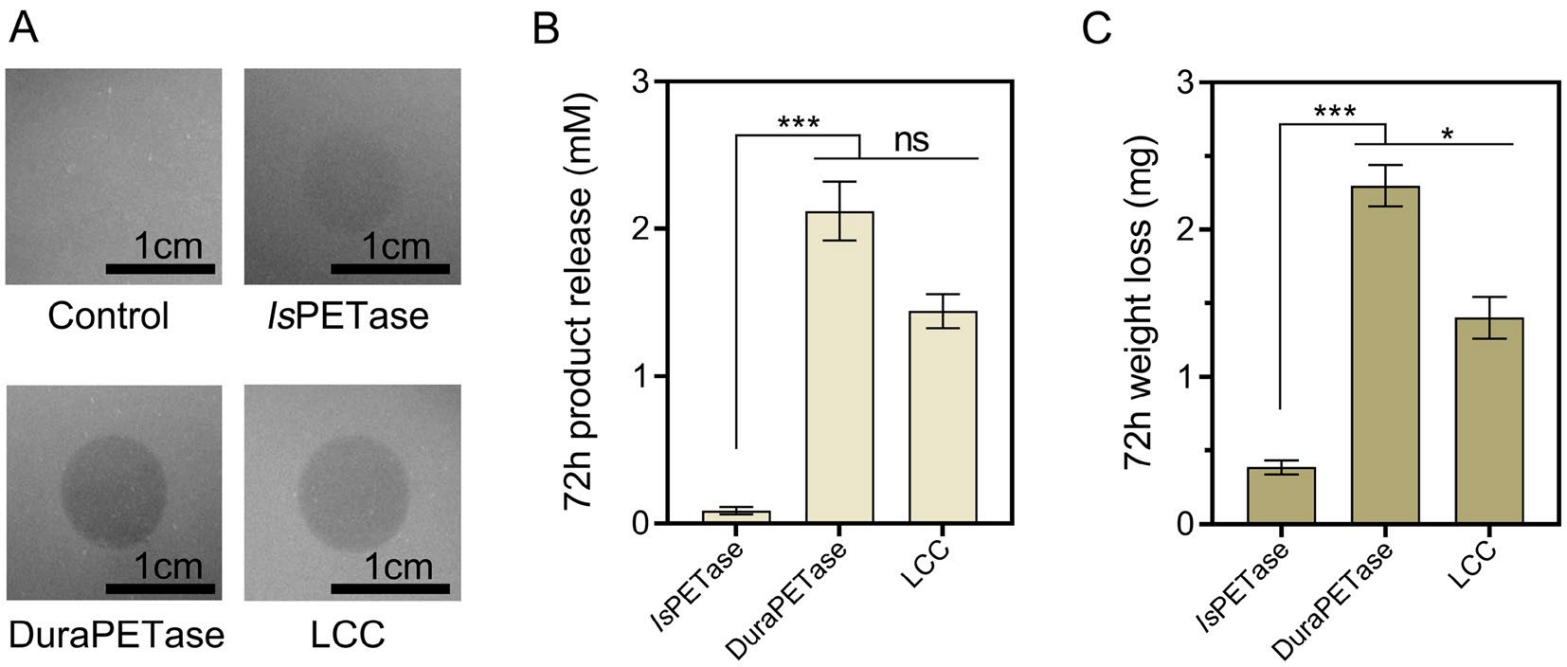
Comparison of hydrolytic activity was conducted among *Is*PETase, DuraPETase, and LCC at a temperature of 30 °C. **A** Transparent zones were formed through the incubation of purified enzymes with Impranil® DLN as the substrate. **B** The release of degrading products from PET films after 72 h of incubation with *Is*PETase, LCC, and DuraPETase at 30 °C was analyzed. The total product release was quantified by summing the detected released compounds (TPA, MHET, and BHET). **C** The weight loss of PET films after incubation with purified enzymes for 72 h, with 100 mg PET films being utilized for degradation

We first purified the three enzymes and tested their activities by observing transparent zones on Impranil^®^ DLN plates (Brott et al. 2022). After dropping with the same amount of enzymes and incubating for 2 h, DuraPETase showed a clearer zone than *Is*PETase and LCC (Fig. 1A), indicating that DuraPETase exhibited higher catalytic activity at 30 °C. To further confirm this, we measured the depolymerization efficacy of the three enzymes using PET films (Goodfellow) as a substrate. After treating the films with an equal amount of enzymes, we monitored the weight loss and the total products released from the PET films. The total released products (TPA, MHET, and BHET), determined by HPLC indicated that the highest degradation product release was observed for DuraPETase (Fig. 1B), which is about 1.5 folds than that of LCC, and over 10 times higher than that of *Is*PETase (Fig. 1B). These data also supported the weight loss of the PET films (without pretreatment) after enzymatic degradation. After 72 hours of enzymatic treatment, DuraPETase resulted in a weight loss of 2.2 mg (Fig. 1C), whereas *Is*PETase and LCC only showed weight losses of 0.4 mg and 1.4 mg, respectively (Fig. 1C). Overall, these results indicated that DuraPETase exhibited the highest catalytic activity at 30 °C, and is a suitable enzyme for subsequent heterologous expression in *C. testosteroni* CNB-1.

### Functional expression of DuraPETase enabled PET degradation by *C. testosteroni* CNB-1

**Fig. 2:**
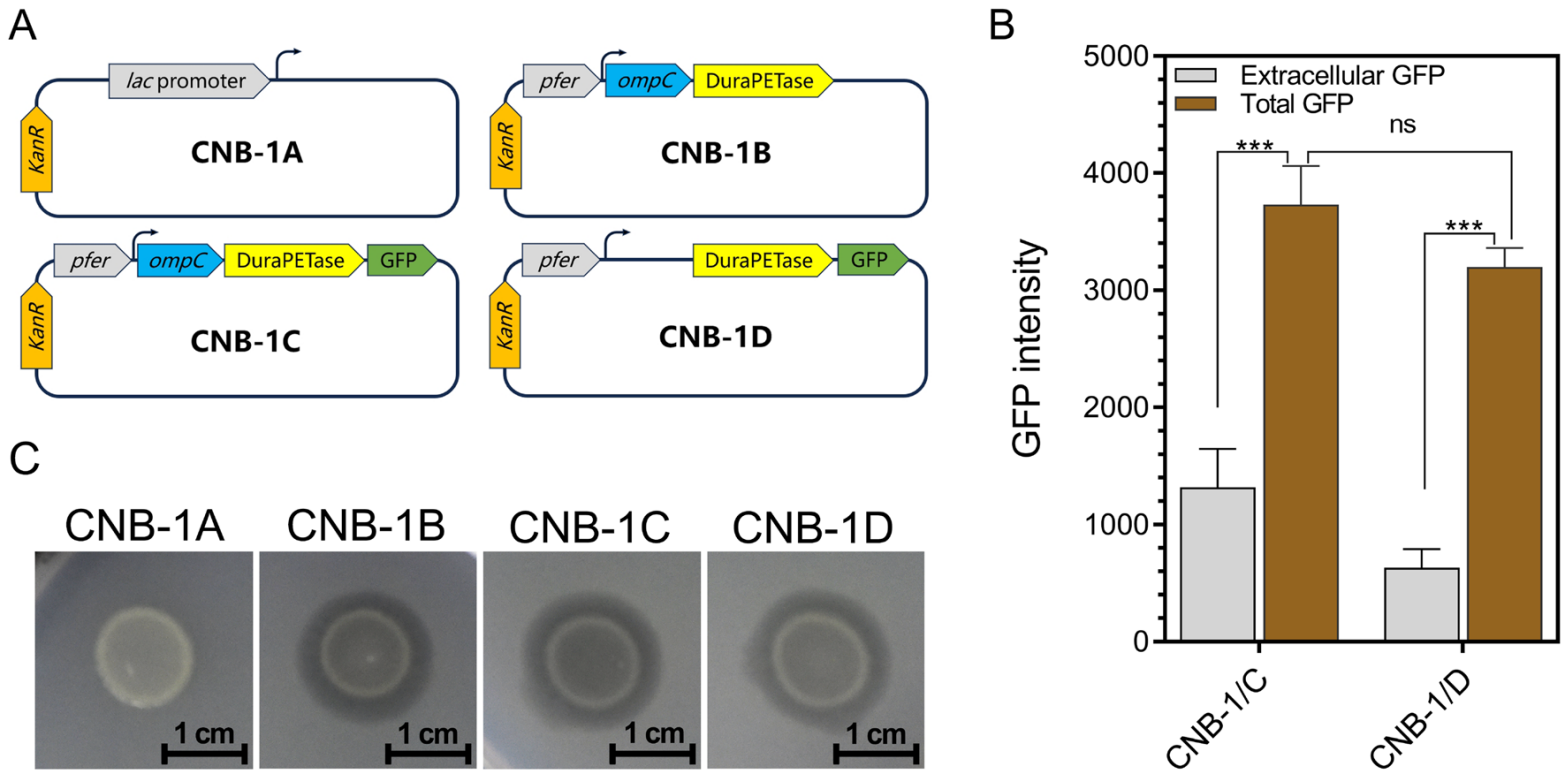
Heterogeneous expression of DuraPETase was observed in *C. testosteroni* CNB-1. **A** DuraPETase was fused with different signal peptide and promoters to facilitate extracellular secretion in CNB-1. Different recombinant plasmids were constructed and introduced into CNB-1 to generate corresponding strains, which were designated as CNB-1A to CNB-1D, respectively. **B** The secretion efficiency of DuraPETase with or without OmpC signal peptide, was determined by measuring the intensity of extracellular GFP, which was fused with DuraPETase. **C** The hydrolytic activity of the recombinant strains was assessed on Impranil^®^ DLN plates

To efficiently degrade PET, the secretion of DuraPETase is crucial, as PET is a high molecular weight polymer that cannot enter cells. To achieve this, we selected a native signal peptide from CNB-1, named OmpC (gene locus: CtCNB1_1477), to assist the efficient secretion of DuraPETase in CNB-1. OmpC is a frequently used signal peptide for efficient secretion of heterologous proteins (Zhou et al. 2018). In addition to extracellular secretion, a high level of DuraPETase transcription is also essential for PET degradation. To accomplish this, a strong constitutive promoter from *C. testosteroni* CNB-1 to replace the promoter in the pBBR1MCS-2 plasmid, resulting in a new plasmid called pBBR1MCS2pfer was used (Huang et al. 2019). The signal peptide and the DuraPETase gene were inserted into the pBBR1MCS2pfer. Then this recombinant plasmid was transformed into *C. testosteroni*, generating the CNB-1B strain. Similarly, we also constructed a recombinant strain containing the DuraPETase gene with GFP, a strain without the signal peptide, and a strain with the original empty plasmid (Fig. 2A).

We chose CNB-1C (with SP_OmpC_) and CNB-1D (without SP_OmpC_) to test the secretion efficiency of the recombinant strains. Both of these strains are tagged with GFP, and the only difference between them is the signal peptide. As shown in Fig. 2B, OmpC signal peptides (SP_OmpC_) successfully direct the secretion of DuraPETase (Fig. 2B), resulting in a two-fold increase in GFP intensity in the supernatants than the recombinant strain CNB-1D (Fig. 2B). Interestingly, even when no signal peptide was fused, the strain CNB-1D was still able to export extracellular DuraPETase-GFP, although the GFP intensity was much lower than in CNB-1C (Fig. 2B). This could be due to the phospholipid hydrolase activity of DuraPETase. Phospholipid is an important component of the cell membrane, and hydrolysis of phospholipids can enhance membrane permeability and release of DuraPETase into the extracellular milieu (Su et al. 2013). Similar phenomena have been observed in many PET-degrading cutinases expressed in *E. coli* in previous studies (Su et al. 2013).

Also, CNB-1C generates a higher total GFP than CNB-1D, indicating that the signal peptide may contribute to the expression of DuraPETase (Fig. 2B). This is reasonable considering that high concentrations of DuraPETase can be toxic to microbial cells, and a secretion system may reduce cytotoxicity and facilitate higher-level expression of DuraPETase. Furthermore, when using Impranil^®^ DLN as a substrate, all the recombinant strains showed similar transparent zones except CNB-1A, which only contains an empty vector (Fig. 2C). This is consistent with the extracellular GFP intensity data, as DuraPETase can be successfully secreted when no signal peptide was fused. Taken together, these data suggested that DuraPETase was functionally expressed in *C. testosteroni* CNB-1, and the signal peptide SP_OmpC_ contributed to the secretion.

### Hydrolytic activity of secreted DuraPETase on PET

**Fig. 3:**
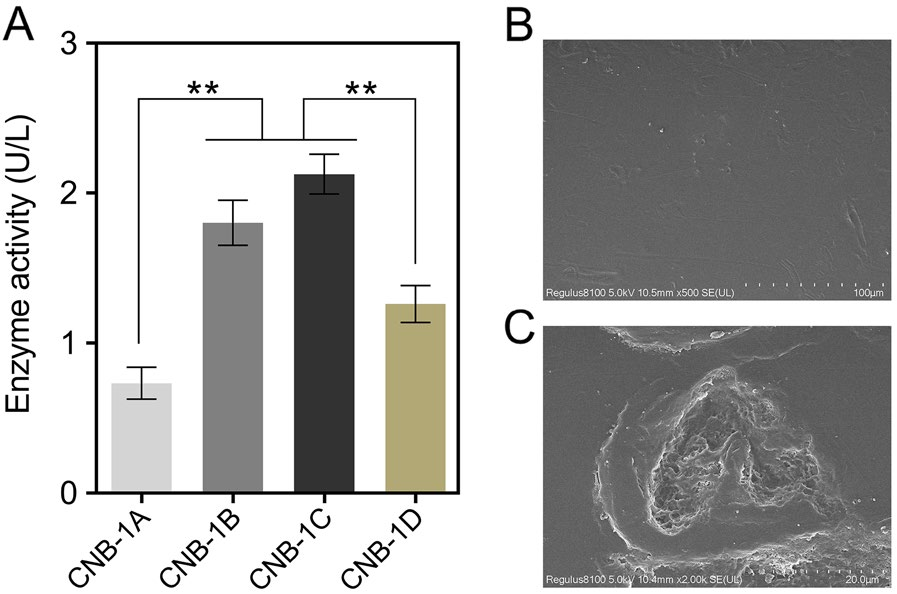
Enzymatic activity analysis was conducted on recombinant strains derived from CNB-1. **A** The catalyst activity of the secreted DuraPETase was determined using *p*-NPB as a substrate. The strains were incubated with MSM medium at 30 °C for 3 days, and 1 mL of the supernatant was collected for incubation with *p*-NPB at 30 °C for 30 min, and the absorbance at 410 nm was measured. SEM images revealed surface erosion of PET films after 14 days of degradation by CNB-1A (**B**) and CNB-1B (**C**), at 30 °C

To investigate whether the DuraPETase secreted by *C. testosteroni* CNB-1 is active against PET, we conducted enzymatic analysis using model substrates such as *p*-NPB. Although the structure of *p*-NPB is different from PET, it has been reported that the conversion rates of small-molecule para-nitrophenyl esters are comparable to PET oligomers (Wei et al. 2022). Therefore, we first used *p*-NPB as a substrate to assess the hydrolytic activity of DuraPETase when expressed in CNB-1. As expected, CNB-1B and CNB-1C exhibited significant kinetic hydrolytic activity against *p*-NPB compared to the blank control CNB-1A, and CNB-1D (Fig. 3A), another recombinant strain lacking a secretion signal peptide. Among them, CNB-1C showed a little higher hydrolytic activity than CNB-1B, but the difference was not significant (Fig. 3A). Moreover, the SEM images further confirmed that CNB-1B can induce surface erosion to PET films after a two-week incubation period (Fig. 3C), whereas the PET films incubated with the control strain (CNB-1A) maintained a smooth surface (Fig. 3B). Consequently, we selected the recombinant strain CNB-1B for further experiments.

**Fig. 4:**
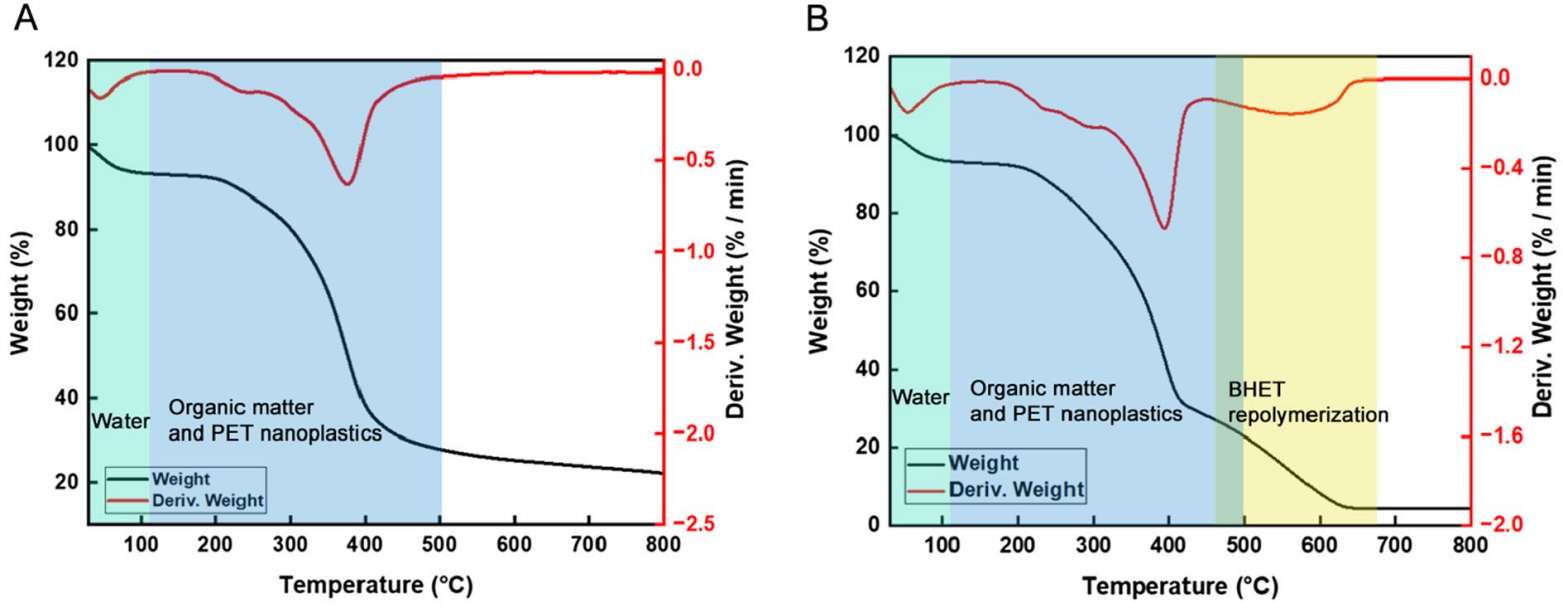
Thermogravimetric analysis (TGA) was conducted to examine the degradation of PET nanoplastics. The differential scanning calorimetry (DSC) and TGA curves of glycolysis products by CNB-1A (**A**) and CNB-1B (**B**) are displayed, with the red line representing the DSC curve and the black line representing the TGA curve. When PET nanoplastics were degraded by the secreted DuraPETase from CNB-1B, the product BHET was released from PET, and the TGA curve showed a distinct BHET polymerization stage, which can be considered as evidence of PET nanoplastics degradation by CNB-1 derived whole-cell biocatalyst

We then investigated the hydrolytic activity of CNB-1B on PET nanoplastics, which were prepared from PET films. These nanoplastics possess a high surface-to-volume ratio, making them significantly more susceptible to hydrolysis in comparison to the films (Barth et al. 2015; Walter et al. 1995). After incubating CNB-1B with PET nanoplastics as the sole carbon source for 10 days, we use thermogravimetric analysis to study the nanoplastics degradation. The thermogram in Fig. 4A illustrates the degradation of nanoplastics by CNB-1 without the heterologous expression of DuraPETase, which shows two stages of weight loss (Fig. 4A). Previous publications have suggested that the first stage of weight loss (approximately 5%) between 25 and 100 °C is caused by the presence of water trapped in nanoplastics and bacterial cells. The second stage of weight loss, occurring between 100 and 500 °C, is attributed to the removal of organic matter from bacterial cells and most of the PET nanoplastics (Fig. 4A). It is important to note that PET decomposes above 250 °C, as indicated by previous reports (Sorolla-Rosario et al. 2022). In comparison, when the nanoplastics are treated with DuraPETase, we observed three distinct stages of weight loss (Fig. 4B). The first two stages are consistent with the findings mentioned above, but the third stage, occurring from 400 to 700 °C (Fig. 4B), is primarily due to the repolymerized of BHET during the heating process in the thermogravimetric study. This observation aligns with a similar thermogram of BHET reported in another study (Scé et al. 2019). These results indicate that PET nanoplastics are degraded by DuraPETase, resulting in the formation of the intermediate compound BHET. This finding suggests that the secreted DuraPETase enzyme is capable of hydrolyzing PET.

### PET microplastic degradation by CNB-1 derived whole-cell biocatalyst

**Fig. 5:**
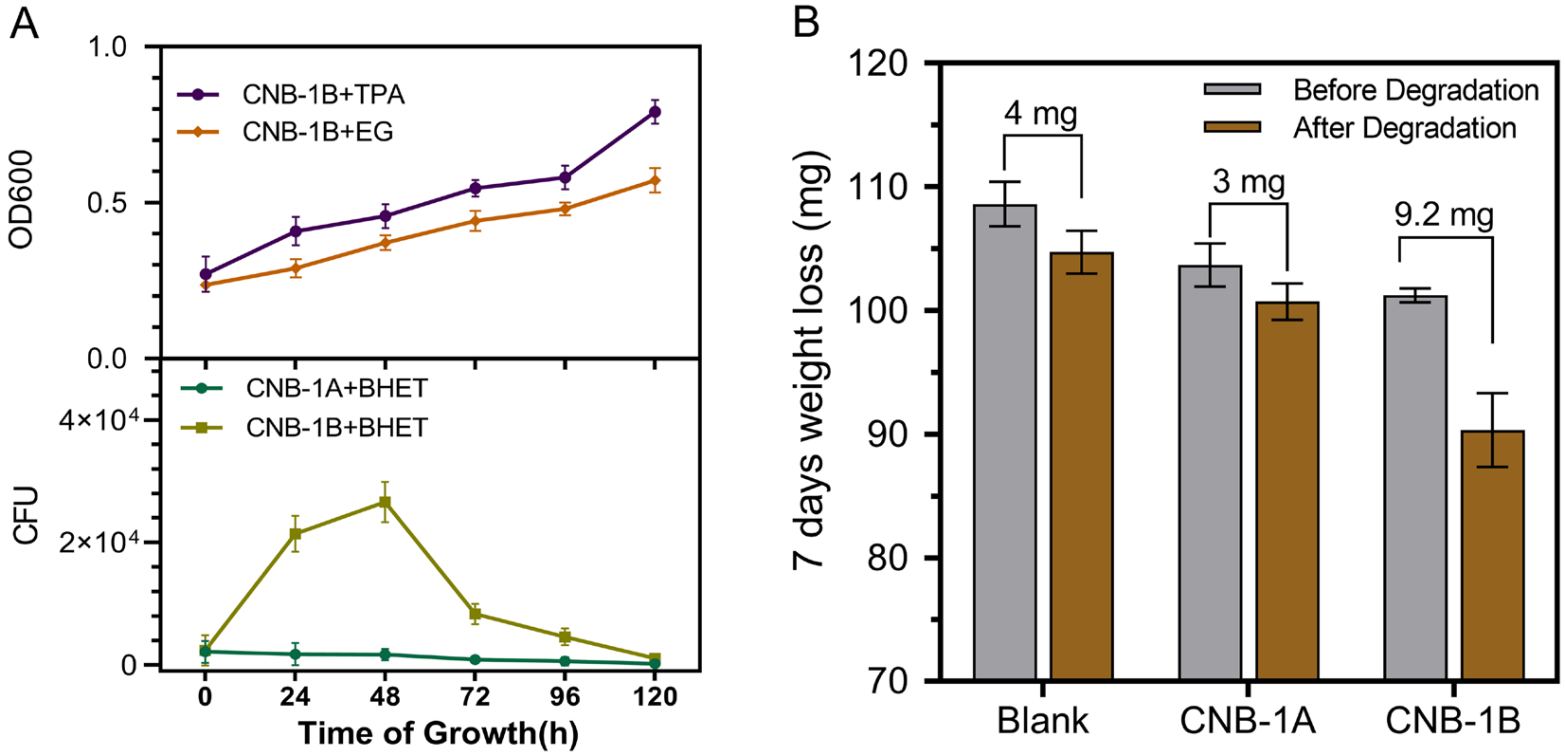
The performance of the CNB-1 derived whole-cell biocatalyst in PET microplastic degradation. **A** The growth curve of CNB-1B using TPA, EG, and BHET as the sole carbon sources. The cell density was measured for TPA and EG, as these compounds are easily utilized by CNB-1B. For BHET, the colony-forming units (CFU) was counted. **B** The degradation of PET microplastics by CNB-1B, 100 mg of PET microplastics was incubated with CNB-1B in MSM + 5% LB (v/v) as a carbon source. For blank control, no carbon source was added. The incubation took place at 30 °C for 7 days

Wastewater treatment plants play a significant role in the entry of microplastics into the environment (Murphy et al. 2016; Sun et al. 2019). It is crucial to develop technology that specifically targets and treats microplastics. Microplastics are primarily found in sludge, which makes separating them difficult and inefficient (Talvitie et al. 2017). Therefore, we have proposed an in situ degradation framework in which engineered *C. testosteroni* CNB-1 is introduced to secrete DuraPETase to hydrolyze microplastics (PET films with an initial crystallinity of approximately 4%). To this end, we first examined the degradation of PET microplastics by the CNB-1 derived PET-degrading whole-cell biocatalyst. After incubating the biocatalysts in MSM medium with 5% (v/v) LB for 7 days, we found that the whole-cell biocatalysts degraded 9.2 mg of the microplastics, which corresponds to a 9% degradation of the total microplastics added (Fig. 5B). This result suggests that when DuraPETase is secreted, the CNB-1 derived whole-cell biocatalyst is capable of degrading PET microplastics.

More importantly, we also found that the CNB-1 derived PET-degrading whole-cell biocatalyst can utilize main intermediates of enzymatic PET degradation, including TPA, EG, and BHET, as sole carbon sources for growth (Fig. 5A). While *Comamonas* is commonly recognized as capable of degrading TPA (Dierkes Robert et al. 2022; Hosaka et al. 2013), the utilization of EG and BHET has not been characterized before. This means that CNB-1-based whole-cell biocatalyst can completely mineralize the PET microplastics, rather than merely degrading it into smaller molecules. In another publication, by functionally immobilizing PETase on the self-assembled *E. coli* curli nanofibers (Zhu et al. 2022), this system could depolymerize highly crystalline post-consumer PET waste materials under ambient conditions with a degradation efficiency of 9.1% in 7 days (Zhu et al. 2022), which is similar to the degradation efficiency observed in this study. Moreover, the whole-cell biocatalyst may have better performance in the real-world sewage sludge system due to the presence of unlimited nutrients (Zhu et al. 2022). This would result in the consumption of organic matter to express depolymerase to hydrolyze microplastics. Our results suggested the potential of CNB-1 derived PET-degrading whole-cell biocatalyst for the removal of PET microplastics in advanced wastewater treatment applications.

With the sludge-indigenous strain *C. testosteroni* CNB-1 as the host, we have proposed an insitu framework for the treatment of sludge microplastics. We have designed a novel whole-cell biocatalyst that facilitates the degradation of PET microplastics by incorporating DuraPETase as the key enzyme, thereby allowing for the efficient depolymerization of PET into TPA and EG. These released intermediates can be further utilized by CNB-1 as the sole carbon source. Thus, the PET-degrading whole-cell biocatalyst derived from CNB-1 opens up a promising approach that could potentially lead to the complete mineralization of PET microplastics present in the sewage sludge. It is worth noting that the microplastic degradation carried out in this study was conducted under small-scale lab conditions. To facilitate the practical application of the PET-degrading whole-cell biocatalyst derived from CNB-1, further research is necessary to evaluate its performance in the degradation of PET microplastics in larger-scale systems such as wastewater treatment bioreactors.

## Conclusion

In this study, we developed a novel whole-cell biocatalyst for the potential removal of microplastics in situ, using sludge-derived *C. testosteroni* CNB-1. We compared the catalytic activity of the reported PET hydrolyses (*Is*PETase, LCC, and DuraPETase) at 30 °C and chose DuaPETase for heterologous expression in *C. testosteroni* CNB-1. After optimizing the promoter and signal peptide, the DuraPETase was successfully secreted into the extracellular milieu from *C. testosteroni* CNB-1. The secretion of DuraPETase led to an efficient hydrolytic activity towards PET nanoplastics and microplastics.

## Data Availability

Data may be made available on request.
